# Optimization and purification of natural protein extract from hazelnut press cake and its antioxidant activity

**DOI:** 10.3389/fnut.2025.1636534

**Published:** 2025-09-09

**Authors:** Yuan Fang, Jiajie Li, Xuyao Zhu, Xinru Li, Songzhe Xu, Hao Wu, Huifeng Zhang, Yanan Luo

**Affiliations:** School of Laboratory Medicine (Pharmaceutical Sciences), Jilin Medical University, Jilin City, Jilin, China

**Keywords:** hazelnut, antioxidant activity, purification, natural protein, optimization

## Abstract

This study aimed to enhance the basic criteria for protein extraction from hazelnuts via the response surface technique and to explore the antioxidant characteristics of the isolated hazelnut protein. The purified hazelnut protein with high quality was obtained using the ÄKTA pure system through a HiTrap DEAE FF column. The refined parameters for extraction included: an extraction duration of 2 h, a temperature set at 33 °C, a liquid-to-solid ratio 8:1, and a 2.32 g protein extraction yield/50 g hazelnut press cake. The crude hazelnut protein was identified by LC-MS, which primarily included 41 proteins (only 10 shown). Antioxidant-activity investigations revealed that purified hazelnut protein had high scavenging activity of DPPH radicals. Furthermore, H_2_O_2_-induced cell oxidative damage was reduced by increasing cell viability and reducing ROS levels. The basis for purified hazelnut protein extraction was also explored. Our findings showed that hazelnut protein may be a promising natural antioxidant.

## 1 Introduction

Typically produced in aerobic metabolism, reactive oxygen species (ROS) are naturally neutralized by the body’s antioxidant mechanism. This delicate equilibrium within cells, between ROS and endogenous antioxidants, is crucial for safeguarding cells from damage. Oxidative stress arises from an imbalance between ROS generation and the body’s protection against antioxidants (1), potentially leading to cellular damage and a range of pathological conditions. When the critical equilibrium of biochemical processes within an organism is disrupted, oxidative stress manifests, inducing an overproduction of ROS. In turn, this ROS surplus induces lipid peroxidation, resulting in DNA and RNA destruction, thereby contributing to metabolic dysfunction (2). Excessive ROS production can lead to harmful effects on cellular organelles and molecules, such as lipids, proteins, and so on (3). Accumulation of cell damage results in dysfunction, culminating in various health issues like aging, diabetes, inflammation, and liver dysfunction (4, 5). The role of antioxidants is both significant and effective. Although many synthetic anti-oxidant drugs are available, they often have side effects that are not viable for long-term use. Additionally, ROS-induced lipid peroxidation can result in the degradation of lipids within cosmetic formulations, thereby potentially compromising the efficacy of these products. Oxidation triggered by free radicals has a direct impact on the quality of food, changing its flavor and texture and reducing its shelf life (6). Utilizing antioxidants and free-radical scavengers to control ROS damage levels shows potential in treating diseases and preventing spoilage in food and cosmetic products. Synthetic antioxidants like propylgallate and butylhydroxyanisole are commonly used, but they come with limitations owing to potential health hazards associated with their use (7). Investigating secure and organic antioxidants that can substitute synthetic substances is a significant matter. Researchers are increasingly focusing on the purification and identification of natural antioxidants. Natural antioxidants tend to be less detrimental than chemically produced ones, whereas compounds like polyphenols, vitamins, polysaccharides, and peptides demonstrate strong antioxidant properties. The exploration of natural antioxidants derived from plant proteins, notably natural protein hydrolysates and antioxidant peptides, has recently gained significant attention and become a key area of focus in research.

Hazelnuts, belonging to Betulaceae family, are highly nutritious and represent an essential source of processed food products. These nuts may be consumed in their raw and cooked forms. Hazelnuts are rich in various important compounds that are beneficial for human dietary health, including polyphenols, proteins, fatty acids, carbohydrates, dietary fiber, aminophenols, and trace elements. While 60% of the dry weight is made up of edible oil, protein accounts for 15% of the overall dry weight (8). For centuries, hazelnuts have been predominantly utilized for the extraction of oil and as a valuable food source. Hazelnut press cake (containing approximately 58% protein) is a significant by-product of oil production (9). It is commonly utilized as animal feed or as a soil fertilizer (10). As the hazelnut processing industry rapidly develops, the inability to recycle and utilize nutritious hazelnut by-products has become a major obstacle to deep processing. It has also created environmental issues for developing countries. Utilizing biotechnology to transform by-product materials into valuable functional components is an effective strategy. Hazelnut press cake is rich in proteins. Currently, reports on the extraction and purification of proteins from hazelnut press cake are relatively few. Exploring the biological activity of proteins in hazelnut press cake is significant. By fully utilizing these proteins, products can be developed, the added value of by-products can be enhanced, and the currently low utilization rate of hazelnut press cake can be improved.

The response surface method (RSM) stands out as a powerful statistical tool for refining and optimizing various processes (11, 12). Meanwhile, the Box–Behnken Design (BBD) offers a structured approach to experimental planning, enabling analysis of experimental outcomes and understand them. In the present study, we used two techniques to isolate protein from hazelnut press cake. One was alkaline dissolution followed by acid precipitation, and the other was ultrasound-assisted extraction. Isolated hazelnut protein was identified by LC-MS, and the crude hazelnut protein was purified with the ÄKTA pure system using HiTrap DEAE FF column. A study focusing on the antioxidant characteristics of processed hazelnut press cake protein laid the groundwork for its creation and usage.

## 2 Materials and methods

### 2.1 Materials

**FIGURE 1:**
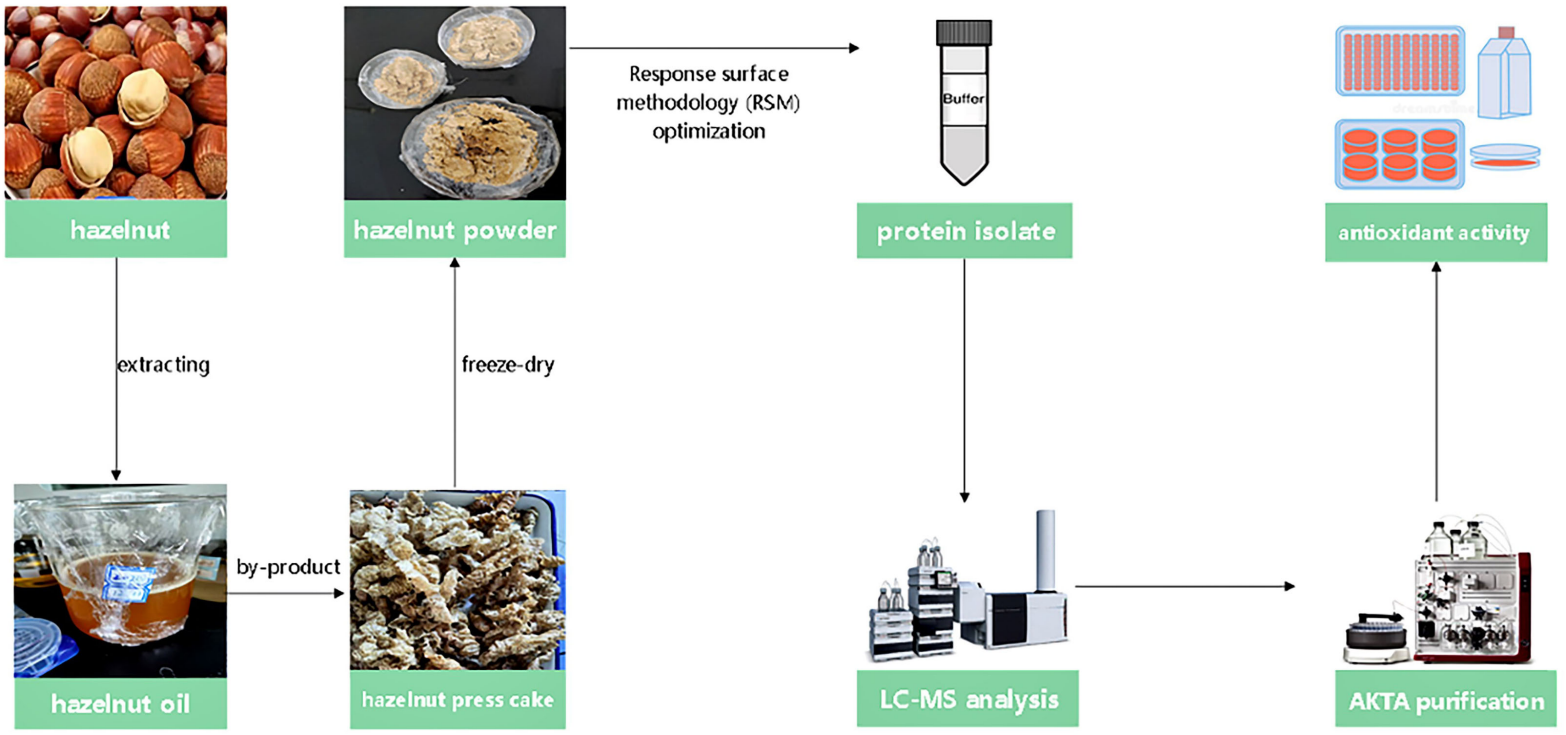
The route map of purified hazelnut protein in this research.

Hazelnuts were available for purchase in a market in Jilin City, Jilin Province. The chemicals and solvents employed were of analytical quality. The flowchart of experimental methods is shown in Figure 1.

### 2.2 Protein extraction

After extracting oil from hazelnuts, the resultant by-product hazelnut press cake was obtained. The hazelnut press cake underwent freeze-drying and was preserved in the fridge at 4 °C before being utilized. Protein extraction from hazelnut press cake involved the use of both alkaline extraction and isoelectric precipitation methods (13). In a typical procedure, 50 g hazelnut press cake was dispersed with distilled water in appropriate proportions. The dispersion pH was raised to 11 using a 1 M sodium hydroxide solution. Stirring and mixing the dispersion for an appropriate amount of time at 20 °C–60 °C ensured that the protein was fully dissolved. Following this, the blend underwent centrifugation at 4,000 *g* for 15 min at ambient temperature to isolate the soluble protein portion from any non-soluble contaminants. Following the separation of the soluble part of the supernatant, the solution’s pH was adjusted to 4.5 using a 1 M hydrochloric acid (HCl) solution to facilitate protein precipitation. Subsequently, the blend underwent centrifugation at 4,000 *g* for half an hour to isolate the protein sediment. The sediment was meticulously extracted from the centrifuge tube, chilled, and measured for its weight. To confirm the exact protein content, the obtained freeze-dried hazelnut protein powder was dissolved in 10-fold Tris solution at pH 10 and subjected to ultrasonic fragmentation for 10 min. Protein quantities were measured utilizing a Biosharp BCA assay kit.

### 2.3 Optimization of hazelnut protein extraction by RSM

Utilizing both alkaline extraction and isoelectric precipitation techniques, a unique factor test was performed to ascertain the ideal conditions for extracting hazelnut protein, the following factors were examined: (1) extraction time (X_1_) range: 0.5–2.5 h; (2) solid-to-liquid ratio (X_2_) range: 1:2 to 1:10; and (3) temperature (X_3_) range: 20 °C–60 °C. Only a single variable was altered in the experiment, and the others remained constant. Protein yield was measured to evaluate the effectiveness of different extraction conditions.

A total of 17 experimental sites with three factors and three levels were established using software for statistical analysis. Utilizing the BBD alongside single-factor experiment outcomes, the procedure of extraction was optimized further using RSM (14, 15). Chosen independent variables included temperature, the time taken for extraction, and the ratio of solid to liquid. The quality of hazelnut protein extraction was evaluated as the response variable in the experimental design. Typically, the foundation of the second-order polynomial model lies in the following Equation 1:


(1) $${\text{Y}}_{0}=a\beta_{0}+\sum_{\text{i}=1}^{3}\beta_{\text{i}}{\text{X}}_{\text{i}}+\sum_{\text{i}=1}^{3}\beta_{\text{ii}}{\text{X}}_{\text{i}}^{2}+\sum_{\text{i}<\text{j}}^{3}\beta_{\text{ij}}{\text{X}}_{\text{i}}{\text{X}}_{\text{j}}+\varepsilon$$

The objective of the model is to verify the impact of each separate factor on a specific reaction. In this equation, Y_0_ is the predicted extraction quality of hazelnut protein, ε is the random experimental error, and *β_0_* and *β_*i*_* are the constant coefficient and the first-order of *X*_*i*_, respectively. In this context, the parameters *β_*ii*_* and *β_*ij*_* denote the quadratic coefficients associated with the variable *X*_*i*_ and the effects of interaction, respectively. *X*_*i*_ and *X*_*j*_ represent the different independent factors.

### 2.4 Identification of hazelnut protein by LC-MS

The crude proteins isolated from hazelnut press cake were analyzed by SDS-PAGE with 12% acrylamide. Electrophoresis was stopped as soon as the sample entered the separation gel. Subsequently, the gel was subjected to CBB staining. The gel containing proteins was divided into 1 mm^3^ fractions. The fractions were dispatched to Beijing and analyzed using LC-MS by Beijing BiotechPack Scientific (Beijing, China) (16). The examination was conducted thrice. The gel particles were decolorized using solution (50% ACN-50% 50 mM NH_4_HCO_3_) until the blue color disappeared. After dissolving the gel particles in 1% DTT solution, the gel particles were digested with trypsin (1.25 μg, diluted by 50 mM NH_4_HCO_3_) at 37 °C for 16 h. Following the initial analysis, the sample was subjected to two rounds of extraction by using 100 L of buffer solution comprising 5% trifluoroacetic acid, 50% acetonitrile, and 45% water. This process was performed at a regulated temperature of 37 °C, maintained consistently over the course of 1 h to ensure optimal conditions. After the solution underwent sonication and centrifugation, the extracted substances were merged and then vacuum-dried. Subsequently, the peptides underwent separation via Acclaim PepMap and Acclaim PepMap RSLC C18 columns. The peptides were eluted at a speed of 600 NL every minute. The gradient process proceeded in this manner: 0–2 min using 4%–8% buffer B; 2–35 min with 8%–28% buffer B; 35–55 min with 28%–40% buffer B; and 55–66 min with 40%–95% buffer B. Buffer A was composed of 0.1% formic acid (CH_2_O_2_), whereas buffer B included 0.1% formic acid (CH_2_O_2_) mixed with 80% acetonitrile. The initial LC-MS files were examined and compared with the protein database according to the sample types by utilizing MaxQuant (2.4.9.0). Solely peptides that were highly reliable were chosen for the ensuing analysis of protein identification. A protein was considered satisfactorily identified if at least one unique peptide was detected (17).

### 2.5 Hazelnut protein purification

The obtained freeze-dried hazelnut protein powder was dissolved in 10-fold Tris solution at pH 10 and ultrasonically fragmented 10 min. To condense the protein solution, the mixtures were poured into Amicon^®^ Ultra-15 Centrifugal Ultrafiltration tubes and centrifuged at 4,000 *g* under r.t. conditions. This step was repeated several times until the volume of concentrated solution reached 300 μL. A BCA assay kit was employed to determine the protein levels in the enriched mixture with hazelnut protein. Subsequently, the enhanced hazelnut protein solution underwent purification through a HiTrap DEAE FF column equipped with an ÄKTA pure system. Buffers for HiTrap DEAE FF column were (i) 20 mM Tris-HCl (pH10) (A buffer) and (ii) 50 mM Tris-HCl (pH 10), 1 M NaCl (B buffer). The solution collected from the peak tubes was enriched after performing ÄKTA pure system purification. The enriched purified hazelnut protein was stored at 4 °C before use. Protein levels were accurately measured with the dependable BCA Protein Assay kit. To check sample, flow-through fluid collected before running buffer B and concentrated solution collected from peak tubes were performed SDS-PAGE analysis at the same time.

### 2.6 DPPH

The capability of the purified hazelnut protein to scavenge DPPH radicals was verified through an earlier documented alteration technique (18, 19). About 100 μL of DPPH (0.1 mM) ethanol solution was mixed with 100 μL water containing different amounts of purified hazelnut protein (0.05–0.5 mg/mL). After thoroughly agitating the solution, it was allowed to react in darkness for an additional 30 min. The final solution was analyzed at 517 nm by utilizing a spectrophotometer machine. Ascorbic acid (vitamin C) was utilized for comparison in this experiment because of its established efficacy and reliability. The effectiveness of DPPH in neutralizing radicals was determined through the following Equation 2:


(2) $$\mathrm{scavenging}\mathrm{activity}\left(\%\right)=\left[1-\frac{A_{i}-A_{j}}{A_{0}}\right]\times 100\%$$

In this context, A_0_ refers to the blank sample absorbance, A_*j*_ symbolizes the empty absorbance, while A_*i*_ indicates the absorbance level of the active specimen.

### 2.7 Cell culture

The study employed Hek293 cells from passages 10 through 15. The cultivation of Hek293 cells took place in a DMEM medium, enriched with 10% fetal bovine serum and a blend of 1% penicillin-streptomycin. The items were kept in a regulated setting at 37 °C, characterized by 5% CO_2_ and 90% humidity. The purified hazelnut protein was added at the same time as H_2_O_2_.

### 2.8 Measurement of cell viability

The impacts of the purified hazelnut proteins on Hek293 cells were evaluated by a CCK-8 assay kit. Cells were grown in a 96-well plate (5 × 10^4^ cells per well) and subsequently preserved in DMEM with varying H_2_O_2_ levels, optionally including purified hazelnut proteins, for 24 h. After incubation, CCK-8 buffer was added, and the plates were kept in a CO_2_ incubator for approximately 1.5 h. The measurement of absorbance at 450 nm was conducted using a Thermo Fisher Scientific microplate reader. In the experiments described above, each group had three parallel samples. Cell viability was obtained with the following Equation 3:


(3) $$\mathrm{Cell}\mathrm{viability}\left(\%\right)=\left({\text{OD}}_{\text{test}}\text{-}{\mathrm{OD}}_{\text{blank}}\right)/\left({\text{OD}}_{\text{control}}\text{-}{\mathrm{OD}}_{\text{blank}}\right)$$

×100%

### 2.9 Measurement of cellular ROS

The assessment of intracellular ROS levels was conducted with the DCFH-DA fluorescent probe, known for its sensitivity to oxidants (Servicebio). Hek293 cells were kept in CO_2_ incubator for one day with various concentrations of H_2_O_2_ or isolated purified hazelnut proteins. Following the extraction of the medium, DCFH-DA, diluted a 1,000 times, was introduced into a new FBS-free medium and left to incubate at 37 °C for 30 min. The levels of ROS in cells were quantitatively assessed using a flow cytometer following two PBS medium washes (20).

### 2.10 Data processing and statistical analysis

Each experiment was repeated thrice to guarantee its dependability. To enhance comprehension of the findings, the data is presented as an average ± standard deviation. The differences between the any two groups were thoroughly evaluated by ANOVA, a statistical method that enabled the comparison of means across multiple groups. Significantly statistical differences were identified based on the following significance levels: *P < 0.05 indicates a low probability that the observed differences are due to chance; **A *P*-value less than 0.01 indicates a more substantial level of significance; whereas ***P-value less than 0.001 signifies an exceptionally high level of statistical significance. Flow cytometry data were rigorously analyzed with FlowJo software (TreeStar, Ashland, OR, United States), ensuring precision and reliability in the results obtained.

## 3 Results and discussion

### 3.1 Single-factor experimental analysis

**FIGURE 2:**
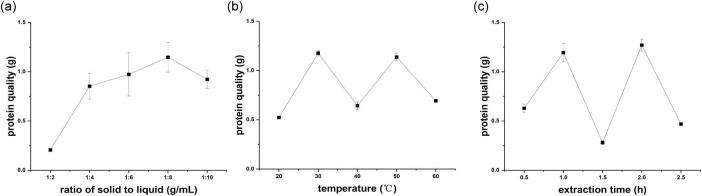
**(a)** Ratio of solid to liquid (g/mL); **(b)** temperature (°C); **(c)** extraction time (h).

The effect of the solid-to-liquid proportion was analyzed in a regulated environment, particularly by employing a 1.5 h extraction duration at 40 °C. The yield of crude hazelnut protein showed a noticeable increase at ratios between 1:2 and 1:8, peaking at a ratio of 1:8 before declining (Figure 2a). The reason may be that the increased ratio of solid-liquid improved the contact between the water and hazelnut sample, thereby improving the yield of crude hazelnut protein. The optimal condition for extraction established was a solid-liquid proportion of 1:8, proven to produce the most efficient outcomes.

The impact of temperature on the yield of crude hazelnut protein was examined, whereas keeping two other extraction parameters constant: an 1.5 h extraction time and a 1:6 solid-liquid ratio. As the temperature rose, the yield of crude hazelnut protein extraction also increased, peaking at 30 °C (Figure 2b). With increased temperature to 40 °C, the extraction yield of crude hazelnut protein decreased.

As the temperature rose to a specific point, the vigorous intermingling of protein molecules disrupted the bonds that held the proteins’ complex structures in place, revealing the hydrophobic protein groups internally. Consequently, the interaction between proteins and water molecules weakened, and protein solubility decreased. With decreased protein solubility, the extraction yield of crude hazelnut protein also decreased. Accordingly, temperature 30 °C was chosen as the best extraction condition.

The impact of extraction time from 0.5 to 2.5 h was determined when the temperature of 40 °C and ratio of solid to liquid of 1:6 were kept unchanged. The highest extraction yield of crude hazelnut protein was observed at an extraction time of 2 h, up to 1.27 g (Figure 2c). Yet, there was a significant reduction in the yield when the extraction duration reached 1.5 h. The possible reason was that the degradation rate of protein may be over the protein dissolution rate, causing transient decreased yield of crude hazelnut protein. Therefore, the extraction time of 2 h was determined to be the most effective condition for optimal results.

### 3.2 RSM optimization

The single-factor study’s results indicated that extraction duration, liquid-to-solid ratio, and temperature were prominent independent variables. In this research, the output of raw hazelnut protein was employed as the dependent variable. Utilizing RSM, seventeen experimental clusters were conducted, integrating three elements across three separate tiers. Comprehensive findings are concisely presented in Tables 1, 2.

**TABLE 1:** Analytical factors and levels of response surface method (RSM).

	Levels		
Factors	−1	0	1
Ratio of liquid to solid (mL/g)	6:1	8:1	10:1
Temperature (°C)	20	30	40
Extraction time (h)	1.5	2	2.5

**TABLE 2:** Program and experimental results of response surface method (RSM).

No.	Extraction time (h)	Ratio of liquid to solid (mL/g)	Temperature (°C)	Protein quality (g)
1	1.5	6:1	30	1.16
2	2.5	6:1	30	1.29
3	1.5	10:1	30	0.76
4	2.5	10:1	30	0.86
5	1.5	8:1	20	0.92
6	2.5	8:1	20	0.85
7	1.5	8:1	40	1
8	2.5	8:1	40	1.43
9	2	6:1	20	1.2
10	2	10:1	20	1
11	2	6:1	40	1.3
12	2	10:1	40	0.94
13	2	8:1	30	2.43
14	2	8:1	30	2.45
15	2	8:1	30	2.55
16	2	8:1	30	2.6
17	2	8:1	30	2.51

The test data were analyzed utilizing quadratic multiple regression techniques to achieve a comprehensive fit. The protein quality response, denoted as Y0, can be estimated using the second-order polynomial (Equation 4):


(4) $$\begin{array}{cc} {\text{Y}}_{0}=-26.44725+11.86150\,{\text{X}}_{1}+2.84912\,{\text{X}}_{2}+0.384400\,{\text{X}}_{3} & \\ -0.007500\,{\text{X}}_{1}{\text{X}}_{2}+0.025000\,{\text{X}}_{1}{\text{X}}_{3}-0.002000\,{\text{X}}_{2}{\text{X}}_{3}-3.10100\,{\text{X}}_{1}^{2} & \\ -0.178813\,{\text{X}}_{2}^{2}-0.006828\,{\text{X}}_{3}^{2} & \end{array}$$

X_1_ signifies the time taken for code extraction, X_2_ indicates the liquid-solid ratio in the code, and X_3_ represent temperature. For the multiple regression model mentioned above, a variance analysis was conducted. The model demonstrated notable importance, evidenced by a substantial F-value (77.25) and a minimal *P*-value (below 0.0001). As depicted in Table 3, the model’s determination coefficient R^2^ stands at 0.9900 and its adjusted R^2^ at 0.9772, signifying a strong correlation between the real and forecasted values. The precision measure of the Adeq (21.2449 > 4) suggested the regression model’s high accuracy. The linear coefficients and the coefficients of the quadratic terms ($X_{1}^{2}$, $X_{2}^{2}$ and $X_{3}^{2}$) significantly affected protein yield (*P* < 0.05). Conversely, the influence of the residual term coefficients was moderately significant (*P* > 0.05). The results revealed the regression model’s remarkable precision in analyzing and forecasting the yield of hazelnut protein extraction.

**TABLE 3:** Analysis of variance (ANOVA) for response surface quadratic model for the yield of protein quality^a^.

Source	Sum of squares	F-value	*P*-value
Model	7.84	77.25	< 0.0001
X_1_	0.0784	6.95	0.0336
X_2_	0.2401	21.29	0.0024
X_3_	0.3496	30.99	0.0008
X_1_X_2_	0.0002	0.0199	0.8917
X_1_X_3_	0.0625	5.54	0.0508
X_2_X_3_	0.0064	0.5674	0.4759
${\text{X}}_{1}^{2}$	2.53	224.36	< 0.0001
${\text{X}}_{2}^{2}$	2.15	190.97	< 0.0001
${\text{X}}_{3}^{2}$	1.96	174.01	< 0.0001
Residual	0.0790	–	–
Lack of fit	0.0593	4.02	0.1063
Pure error	0.0197	–	–
Cor total	7.92	–	–
R-squared	0.9900	Pred R-squared	0.8764
Adj R-squared	0.9772	Adeq precision	21.2449

*^a^*Results were obtained with Design Expert 8.0.6.

**FIGURE 3:**
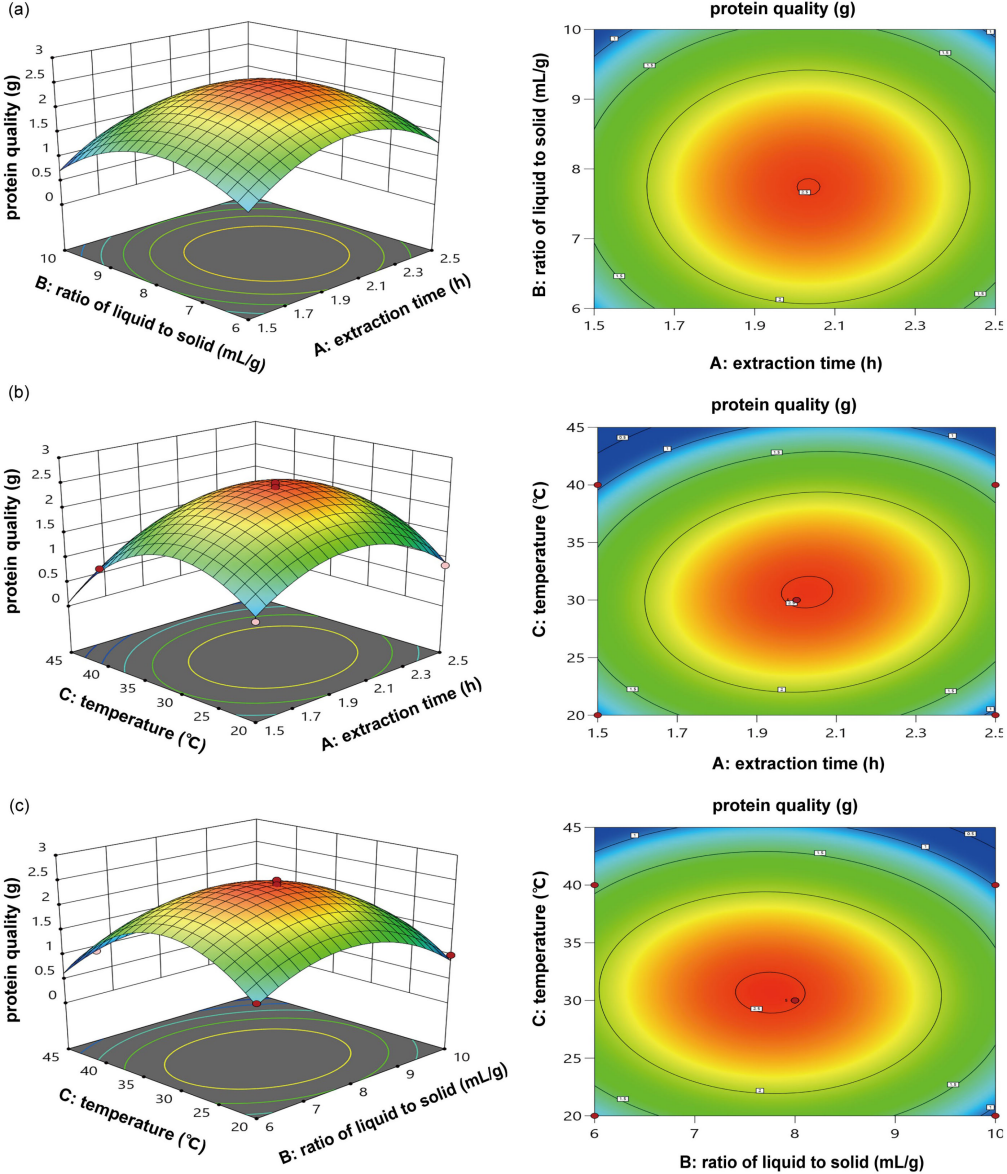
3D response surface and contour diagram of the yield of protein quality; **(a)** extraction time (h) vs. ratio of liquid to solid (mL/g); **(b)** extraction time (h) vs. temperature (°C); **(c)**, temperature (°C) vs. ratio of liquid to solid (mL/g).

Results derived from the contour map and the 3D response surface generated through RSM distinctly showcased the interaction between these two distinct factors. The interplay between the liquid-to-solid ratio and the duration of extraction (Figure 3a), the duration of extraction and temperature (Figure 3b), along with the temperature and the ratio of liquid to solid (Figure 3c), are clarified using a three-dimensional response surface and its related contour map. Enhancing the duration of extraction and the liquid-to-solid ratio improved hazelnut protein production, while extended extraction periods and increased liquid-to-solid ratios resulted in reduced yield (Figure 3a). The protein yield exhibited an increase corresponding with elevated temperatures and extended extraction durations (Figure 3b). The output of raw hazelnut protein corresponded with the findings from experiments involving a single factor. The production capacity initially showed a promising increase with higher temperatures and ratios of liquid to solid, but ultimately experienced a notable decline (Figure 3c).

The ideal extraction parameters derived from Design Expert 13 included: an extraction time of 2 h, a liquid-to-solid ratio of 8:1, and a temperature of 32.5 °C. Under this condition, the forecasted extraction yield of crude hazelnut protein can reach 2.49 g. In accordance with the established experimental parameters, the extraction conditions were carefully adjusted. Specifically, the extraction time was set 2 h, the ratio of liquid to solid was determined 8:1, and the temperature 33 °C was regulated to optimize the process. To effectively validate the process, we conducted three meticulously designed experiments based on the specified conditions. Results indicated an average protein extraction production capacity of 2.32 g. The outcome closely matched the expected value. Therefore, the yield of crude hazelnut protein can be significantly enhanced by optimizing factors such as extraction duration time, liquid-to-solid ratio, and temperature.

### 3.3 LC-MS identification of crude hazelnut protein

Proteins pinpointed through LC-MS are displayed in Table 4. Totally proteins were identified, and only 10 protein names obtaining a high score are shown.

**TABLE 4:** Protein isolated from hazelnut press cake by LC-MS/MS analysis.

Entry name (UniProt)	Protein description^1^	Unique peptides	Intensity (× 10^10^)	Predicted Mw (kDa)^2^	Score
A0A0A0P7E3	Cor a 9 allergen	8	116.39	58.836	323.31
Q8S4P9	Vicilin Cor a 11.0101	35	49.811	50.855	323.31
Q84T91	Oleosin Cor a 13	4	8.6358	14.732	323.31
Q84T21	Oleosin Cor a 12	8	2.5603	16.698	323.31
A0A6C0PBB1	Caleosin 1	14	1.5611	26.815	323.31
Q9FPK3	Major allergen variant Cor a 1.0403	4	0.79969	17.527	323.31
C0HM28	Oleosin Cor a 15	12	4.2676	17.695	246.53
D0PWG2	2S albumin	4	1.0684	17.078	171.07
Q8W1C2	11S globulin-like protein	4	8.9428	59.127	113.69
Q9FSY7	Endoplasmic reticulum chaperone BiP	10	0.73492	73.563	81.557

^1^Protein function was predicted by UniProt (https://www.uniprot.org/).

^2^Molecular weight was predicted by ExPASy-compute pI/Mw (https://web.expasy.org/compute_pi/).

### 3.4 Hazelnut protein purification

**FIGURE 4:**
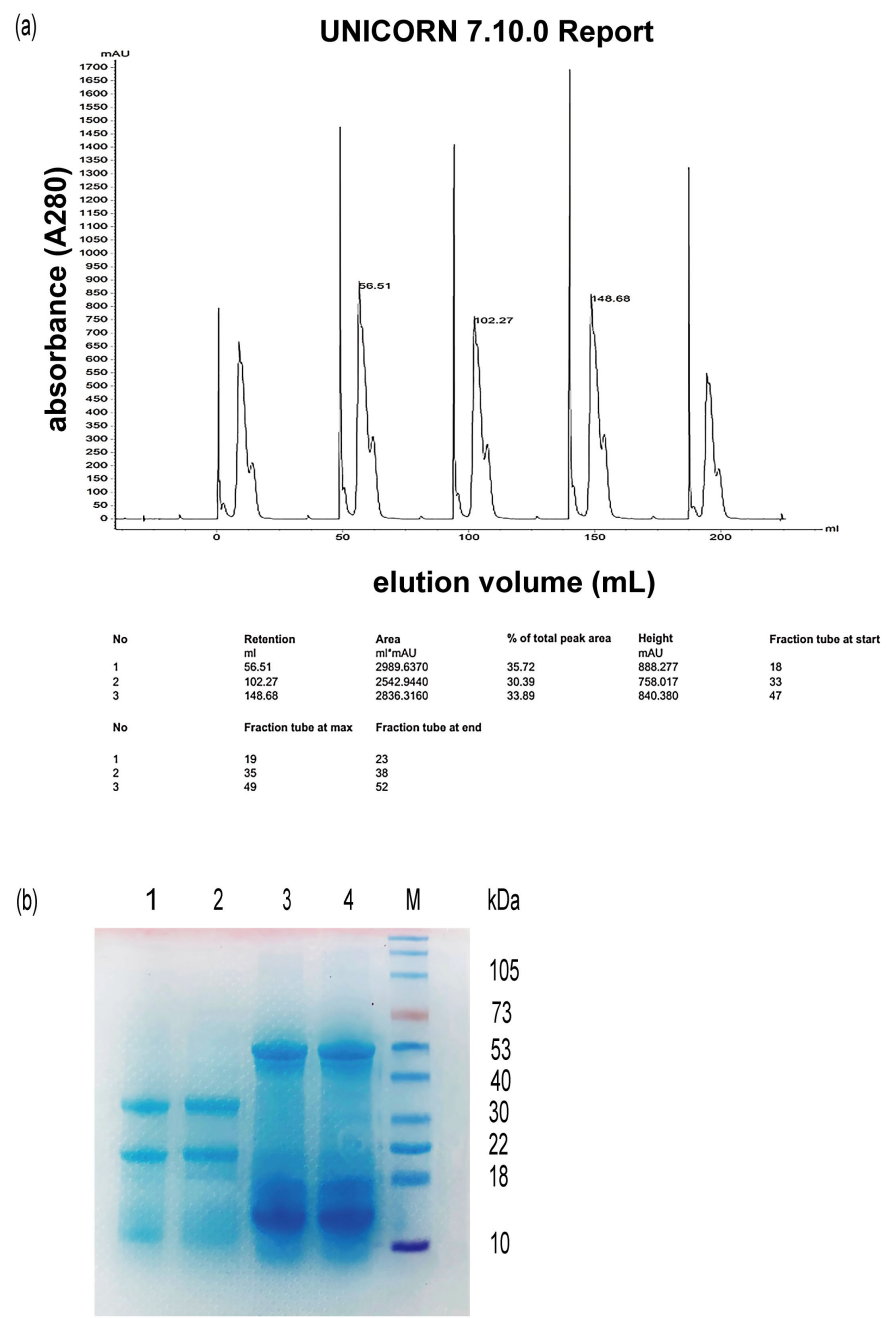
Elution curves of protein isolated from hazelnut press cake on HiTrap DEAE FF column **(a)** and SDS-PAGE analysis **(b)**. **(a)** anion-exchange chromatography analysis of hazelnut protein by HiTrap DEAE FF column; **(b)** SDS-PAGE analysis (M), protein marker, (1) (2), flow-through fluid, (3) (4), concentrated solution collected from peak.

To further identify the antioxidant activity of hazelnut protein, the crude hazelnut protein mixtures were purified using a HiTrap DEAE FF column. About 300 μL of concentrated crude hazelnut protein samples were loaded into the column five times in one experiment, and we obtained enough amount of protein for the subsequent experiments. Elution with buffer A [20 mM Tris-HCl (pH 10)] resulted in the first sharp peak (Figure 4a). Given that the isoelectric point (pI) of various hazelnut proteins differed, some of them can not attach onto the column at pH 10. Elution with buffer B (NaCl solution) resulted in the second wide peak (Figure 4a). The second wide peak fraction solutions were collected and concentrated. Upon confirming the concentration of the purified hazelnut protein through a BCA assay, the purified protein was utilized in subsequent experiments. To check the purified hazelnut protein sample, flow-through fluid and concentrated protein solution collected from the second wide peak were analyzed by SDS-PAGE (Figure 4b). This result was consistent with hypothesis that flow-through fluid contained a small amount of hazelnut protein. The flow-through fluid may contain some impurity substances, so we did not use it for the subsequent antioxidant activity experiments.

### 3.5 DPPH radical-scavenging activity

**FIGURE 5:**
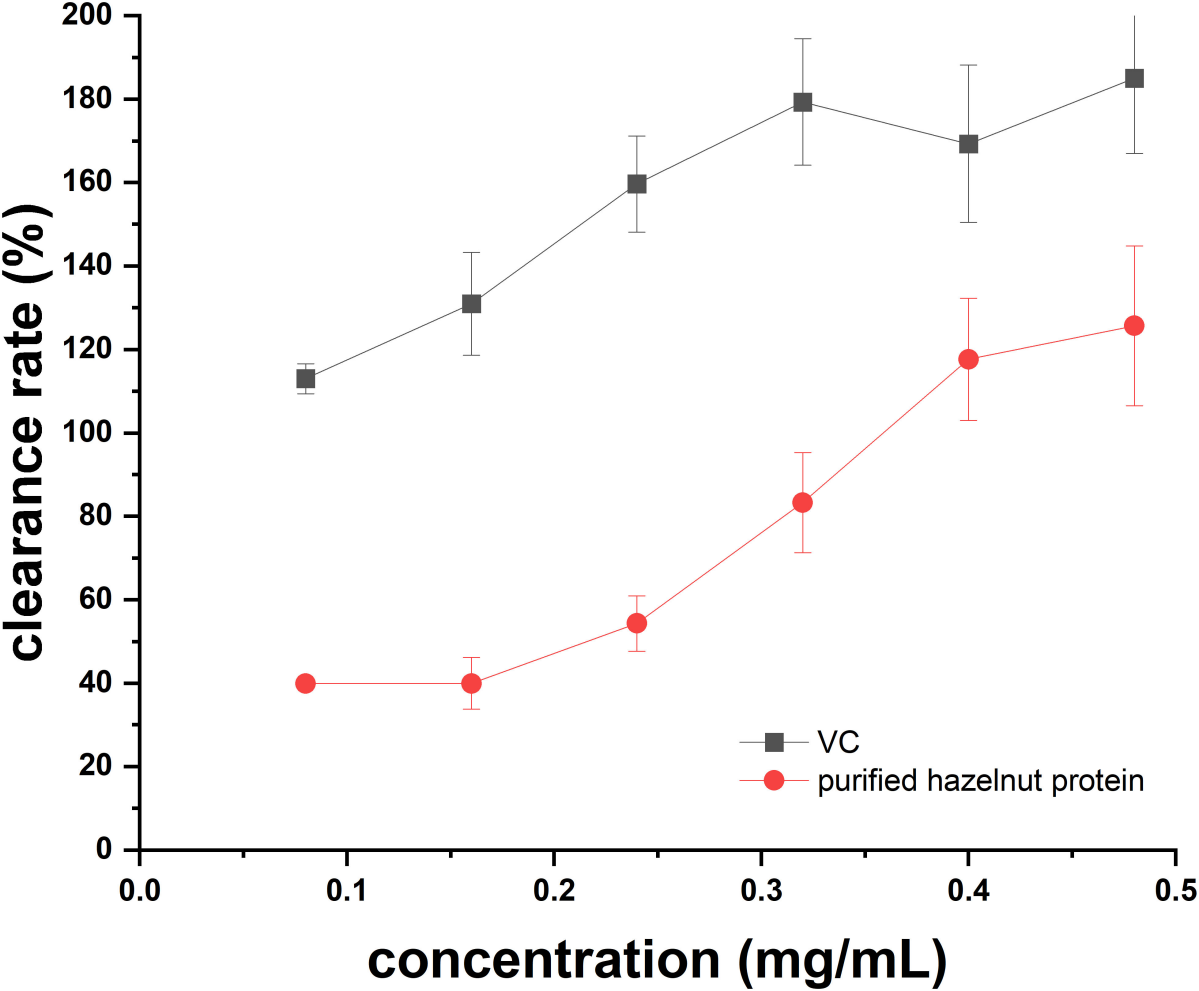
DPPH free radical scavenging ability.

1, 1-Diphenyl-2-picrylhydrazyl (DPPH) is acknowledged for its stability as a free radical and is frequently employed as a dependable technique to assess various antioxidants’ capacity to scavenge free radicals (21). It was evident that the ability of DPPH radicals to scavenge varied with the dosage, falling between 0.08 and 0.48 mg/mL (Figure 5), indicating that the purified hazelnut protein had certain antioxidant activity. However, at each concentration of VC and purified hazelnut protein, the clearance rate of DPPH of VC was higher than that of purified hazelnut protein (Figure 5). These results confirmed that the purified hazelnut protein had a weaker capability of scavenging DPPH radical than VC.

### 3.6 Prevention of H_2_O_2_-induced cell death by purified hazelnut protein

**FIGURE 6:**
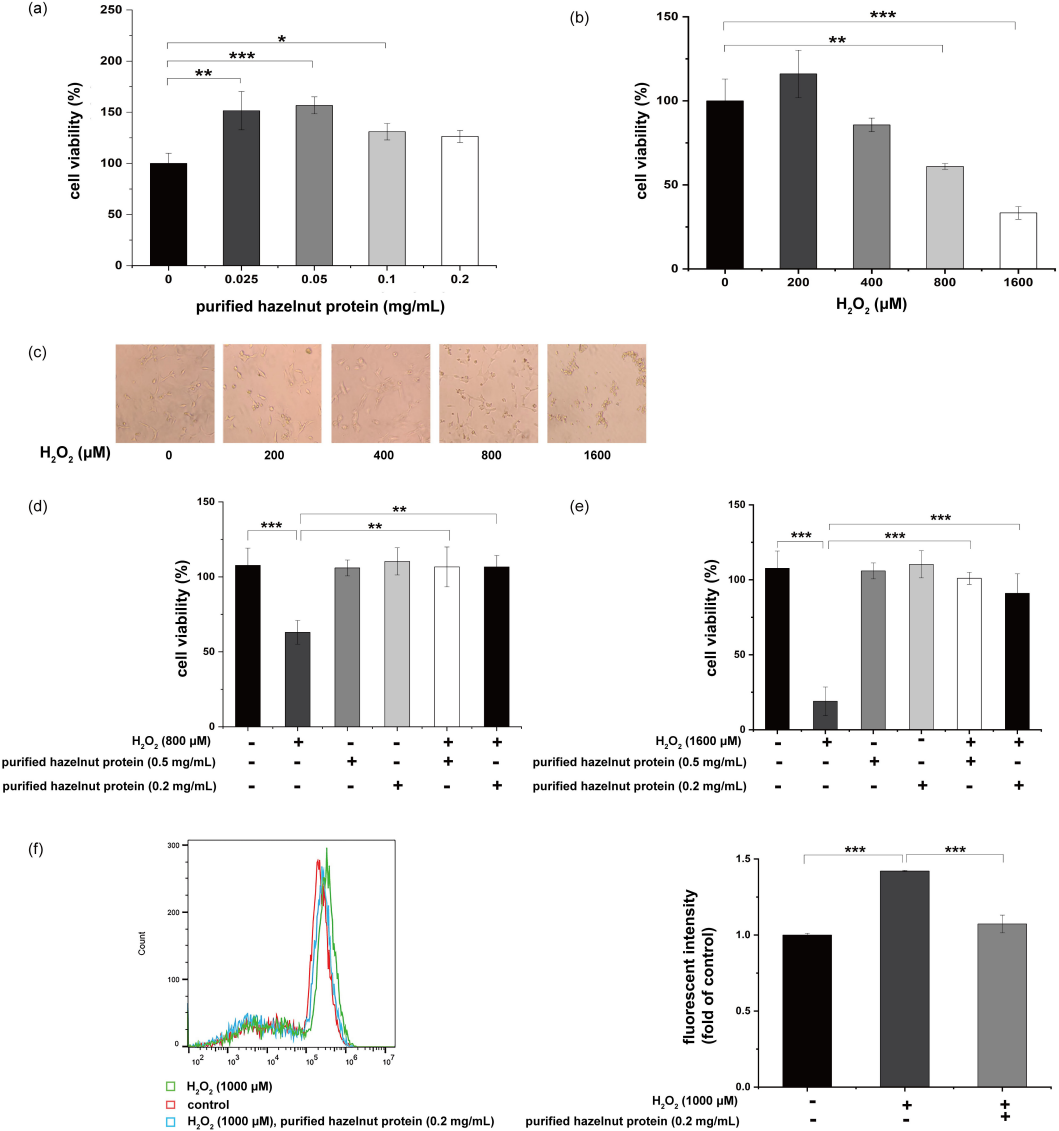
Protective effect by purified hazelnut protein against H_2_O_2_-induced cell death in Hek293 cells. **(a)** Cell viability of purified hazelnut protein in Hek293 cells. Cells were incubated for 48 h with various concentrations of purified hazelnut protein (0.025–0.2 mg/mL; black columns). Cell viability was expressed as values relative to at 0 mg/mL, which was defined as 100%. Data are shown as the mean ± S.D. from three independent experiments. *p ≤ 0.05, **p ≤ 0.01, ***p ≤ 0.001. **(b)** Cell toxicity of H_2_O_2_-treated Hek293 cells. Cells were cultured for 48 h with various concentrations of H_2_O_2_ (100–1,600 μM; black columns). Cell viability was expressed as values relative to at 0 μM, which was defined as 100%. Data represent the mean ± S.D. from three independent experiments. *p ≤ 0.05, **p ≤ 0.01, ***p ≤ 0.001. **(c)** Morphology of H_2_O_2_-treated (100–1,600 μM) Hek293 cells for 48 h. Data are the representative of three independent experiments. **(d)** Protective effect of purified hazelnut protein in H_2_O_2_-treated cells. Hek293 cells were treated for 48 h with various concentrations of purified hazelnut protein (0.2, 0.5 mg/mL) in the presence of H_2_O_2_ (800 μM). Cell viability was expressed as values relative to that of the vehicle-treated cells, which was defined as 100%. Data are presented as the mean ± S.D. from three independent experiments. *p ≤ 0.05, **p ≤ 0.01, ***p ≤ 0.001. **(e)** Protective effect of purified hazelnut protein in H_2_O_2_-treated cells. Hek293 cells were treated for 48 h with various concentrations of purified hazelnut protein (0.2, 0.5 mg/mL) in the presence of H_2_O_2_ (1,600 μM). Cell viability was expressed as values relative to that of the vehicle-treated cells, which was defined as 100%. Data are presented as the mean ± S.D. from three independent experiments. *p ≤ 0.05, **p ≤ 0.01, ***p ≤ 0.001. **(f)** The changes of ROS accumulation in Hek293 cells with the intervention of H_2_O_2_ and purified hazelnut protein measured by flow cytometry.

The cell toxicity of purified hazelnut protein was first investigated in Hek293 cells. No cytotoxic effect was evident up to 0.2 mg/mL purified hazelnut protein. Apparently, purified hazelnut protein slightly stimulated cell growth (Figure 6a). After exposing Hek293 cells to different H_2_O_2_ concentrations (0–1,600 μM) over 24 h, there was a noticeable reduction in cell survival, correlating with the H_2_O_2_ levels applied (Figure 6b). Meanwhile, 200 μM H_2_O_2_ slightly increased cell growth, which may be stress response. The cell results observed by microscopy were nearly identical to the CCK8 results (Figure 6c). The total number of cells was reduced following treatment with H_2_O_2_ (800 μM) (Figure 6d). The decline in cell number caused by the increased levels of H_2_O_2_ was reversed through treatment with purified hazelnut protein (Figure 6d). Moreover, treatment with only purified hazelnut protein did not apparently stimulate cell growth (Figure 6d). The total cell count was noticeably reduced following treatment with H_2_O_2_ (1,600 μM), whereas the addition of purified hazelnut protein at 0.2 or 0.5 mg/mL effectively improved the viability of the cells that had been treated with H_2_O_2_ (Figure 6e). These results indicated that purified hazelnut protein had the ability to protect Hek293 cells against cell death, which was induced by H_2_O_2_. These outcomes revealed that the H_2_O_2_-induced intracellular ROS level was lowered by co-treatment with purified hazelnut protein in Hek293 cells (Figure 6f).

The purified hazelnut protein used in this paper was confirmed to have antioxidant activity. The presence of ROS in cells is vital for various biological functions; however, an excessive accumulation of these molecules can lead to adverse effects when the antioxidant protection system is unable to maintain balance. Excessive load may lead to oxidative harm, a condition linked to the emergence of various diseases (22). Therefore, ROS levels require effective regulation to uphold cellular health and mitigate potential disease risks. Cell models provide a more in-depth understanding of how hazelnut protein influences physiological conditions. However, the mechanisms through which hazelnut protein offers protection against oxidative stress are yet to be explored. Future research should explore how purified hazelnut protein enhances the activity of internal enzymes like superoxide dismutase (SOD), glutathione S-transferase (GST), and catalase (CAT). Fang et al. (23) discovered that hazelnut peptides effectively protect against oxidative stress damage. Specifically, two peptides—EW and DWDPK—were found to inhibit NADPH oxidative activity by lowering NOX2 expression, which in turn mitigated cellular oxidative stress damage. Two transcription factors, Nuclear factor E2-associated protein2 (Nrf-2) and nuclear factor kappa B (NF-κB), play roles in regulating cellular redox reactions. Chronic inflammation is initiated by oxidative stress, while the inflammatory reaction intensifies ROS secretion. The primary mechanism behind this interaction is the NF-κB signaling pathway. Wu and colleagues discovered that the Pro-His-Pro peptide enhances the production of antioxidant enzymes such as CAT and SOD. Furthermore, this led to a rise in Nrf2 levels and a reduction in Keap1 protein levels, which in turn stimulated the transcription of antioxidant response elements mediated by Nrf2 (24).

## 4 Conclusion

Protein was successfully extracted from hazelnut. RSM was employed to improve the conditions for extraction, focusing on improving the liquid-to-solid ratio 8:1 mL/g, the duration of extraction 2 h, and the temperature 33 °C, aiming to maximize the production of raw hazelnut protein. To further accurately identify the activity of hazelnut protein, the ÄKTA purification of hazelnut protein by using HiTrap DEAE FF column was performed. The purified hazelnut protein was found to have remarkably high antioxidant activity. Furthermore, natural antioxidants can serve as viable alternatives to synthetic antioxidants in various applications, including nutritional and health products and therapeutic agents. However, further investigation is essential to enhance our underCstanding of the mechanisms that contribute to their antioxidant properties.

## Data Availability

The authors acknowledge that the data presented in this study must be deposited and made publicly available in an acceptable repository, prior to publication. Frontiers cannot accept a manuscript that does not adhere to our open data policies.

## References

[1] Wang L Park Y Jeon Y Ryu B. Bioactivities of the edible brown seaweed, Undaria pinnatifida: a review. *Aquaculture.* (2018) 495:873–80. 10.1016/j.aquaculture.2018.06.079

[2] Roginskaya M Razskazovskiy Y. Oxidative DNA damage and repair: mechanisms, mutations, and relation to diseases. *Antioxidants.* (2023) 12:1623. 10.3390/antiox12081623 37627618 PMC10451152

[3] Butterfield D Halliwell B. Oxidative stress, dysfunctional glucose metabolism and Alzheimer disease. *Nat Rev Neurosci.* (2019) 20:148–60. 10.1038/s41583-019-0132-6 30737462 PMC9382875

[4] Caro A Davis A Fobare S Horan N Ryan C Schwab C. Antioxidant and pro-oxidant mechanisms of (+) catechin in microsomal CYP2E1-dependent oxidative stress. *Toxicol Vitro.* (2019) 54:1–9. 10.1016/j.tiv.2018.09.001 30195042 PMC6281780

[5] Hancock J Desikan R Neill S. Role of reactive oxygen species in cell signaling pathways. *Biochem Soc Trans.* (2001) 29:345–50. 10.1042/bst0290345 11356180

[6] Nikoo M Benjakul S Xu X. Antioxidant and cryoprotective effects of Amur sturgeon skin gelatin hydrolysate in unwashed fish mince. *Food Chem.* (2015) 181:295–303. 10.1016/j.foodchem.2015.02.095 25794753

[7] Tanzadehpanah H Asoodeh A Chamani J. An antioxidant peptide derived from Ostrich (Struthio Camulus) egg white protein hydrolysates. *Food Res Int.* (2012) 49:105–11. 10.1016/j.foodres.2012.08.022

[8] Jiang J Liang L Ma Q Zhao T. Kernel nutrient composition and antioxidant ability of Corylus spp. in China. *Front. Plant Sci.* (2021) 12:690966. 10.3389/fpls.2021.690966 34249062 PMC8261296

[9] Liu C Fang L Min W Liu J Li H. Exploration of the molecular interactions between angiotensin-I-converting enzyme (ACE) and the inhibitory peptides derived from hazelnut (Corylus heterophylla Fisch.). *Food Chem.* (2018) 245:471–80. 10.1016/j.foodchem.2017.10.095 29287398

[10] Shi C Liu M Zhao H Lv Z Liang L Zhang BL. A novel insight into screening for antioxidant peptides from hazelnut protein: based on the properties of amino acid residues. *Antioxidants.* (2022) 11:127. 10.3390/antiox11010127 35052631 PMC8772696

[11] Zhao F Yan Z Sun J Ma Z Kang X Ji Z Essential oils from citri reticulatae pericarpium: optimization of hydrodistillation extraction and effects of stir-frying processing on chemical components and multiple biological activities. *Industrial Crops Products.* (2022) 189:115825. 10.1016/j.indcrop.2022.115825

[12] Hu Z Wang P Zhou H Li Y. Extraction, characterization and in vitro antioxidant activity of polysaccharides from Carex meyeriana Kunth using different methods. *Int J Biol Macromolecules.* (2018) 120:2155–64. 10.1016/j.ijbiomac.2018.09.125 30248430

[13] Zhang T Jiang B Mu W Wang Z. Emulsifying properties of chickpea protein isolates: influence of pH and NaCl. *Food Hydrocolloids.* (2009) 23:146–52. 10.1016/j.foodhyd.2007.12.005

[14] Tran Q Thi T Do T Thi H Thi B Chu Q Optimization of microwave-assisted extraction process of callicarpa candicans (Burm. f.) Hochr essential oil and its inhibitory properties against some bacteria and cancer cell lines. *Processes.* (2020) 8:173–87. 10.3390/pr8020173

[15] Chen H Bai Z Tao S Li M Jian L Zhang Y Optimization of enzyme-assisted microwave extraction, structural characterization, antioxidant activity and in vitro protective effect against H_2_O_2_-induced damage in HepG2 cells of polysaccharides from roots of Rubus crataegifolius Bunge. *Int J Biol Macromol.* (2024) 276:133969. 10.1016/j.ijbiomac.2024.1.33969 39029849

[16] Talamantes T Ughy B Domonkos I Kis M Gombos Z Prokai L. Label-free LC-MS/MS identification of phosphatidylglycerol-regulated proteins in Synechocystis sp. PCC6803. *Proteomics.* (2014) 14:1053–7. 10.1002/pmic.201300372 24574175

[17] Tyanova S Temu T Cox J. The MaxQuant computational platform for mass spectrometry-based shotgun proteomics. *Nat Protoc.* (2016) 11:2301–19. 10.1038/nprot.2016.136 27809316

[18] Sharma K Assefa A Kim S Ko E Lee E Park S. Evaluation of total phenolics, flavonoids and antioxidant activity of 18 Korean onion cultivars: a comparative study. *J. Sci. Food Agric.* (2013) 94:1521–9. 10.1002/jsfa.6450 24136245

[19] Rumpf J Burger R Schulze M. Statistical evaluation of DPPH, ABTS, FRAP, and Folin-Ciocalteu assays to assess the antioxidant capacity of lignins. *Int J Biol Macromol.* (2023) 233:123470. 10.1016/j.ijbiomac.2023.123470 36736974

[20] Sun W Ni Z Li R Chang X Li W Yang M Flurochloridone induces Sertoli cell apoptosis through ROS-dependent mitochondrial pathway. *Ecotoxicol Environ Saf.* (2021) 216:112183. 10.1016/j.ecoenv.2021.112183 33812209

[21] Li R Chen W Wang W Tian W Zhang X. Extraction, characterization of as tragalus polysaccharides and its immune modulating activities in rats with gastric cancer. *Carbohydr Polym.* (2009) 78:738–42. 10.1016/j.carbpol.2009.06.005

[22] Casas A Nogales C Mucke H Petraina A Cuadrado A Rojo A On the clinical pharmacology of reactive oxygen species. *Pharmacol Rev.* (2020) 72:801–28. 10.1124/pr.120.019422 32859763

[23] Fang L Ren D Wang Z Liu C Wang J Min W. Protective role of hazelnut peptides on oxidative stress injury in humanumbilical vein endothelial cells. *J Food Biochem.* (2018) 43:e12722. 10.1111/jfbc.12722 31353565

[24] Wu J Sun B Luo X Zhao M Zheng F Sun J Cytoprotective effects of a tripeptide from Chinese Baijiu against AAPH-induced oxidative stress in HepG2 cells via Nrf2 signaling. *RSC Adv.* (2018) 8:10898–906. 10.1039/c8ra01162a 35541541 PMC9078957

